# Seroprevalence and Associated Risk Factors of Trichinellosis and *T. Solium* Cysticercosis in Indigenous Pigs in Hoa Binh Province, Vietnam

**DOI:** 10.3390/tropicalmed7040057

**Published:** 2022-04-07

**Authors:** Trang Thi-Huyen Le, Nga Vu-Thi, Sinh Dang-Xuan, Hung Nguyen-Viet, Phuc Pham-Duc, Luong Nguyen-Thanh, Ngoc Pham-Thi, John Noh, Anne Mayer-Scholl, Maximilian Baumann, Diana Meemken, Fred Unger

**Affiliations:** 1 International Livestock Research Institute, Hanoi 100000, Vietnam; t.le@cgiar.org (T.T.-H.L.); h.nguyen@cgiar.org (H.N.-V.); f.unger@cgiar.org (F.U.); 2 National Institute of Veterinary Research, Hanoi 100000, Vietnam; ngancvd@gmail.com (N.V.-T.); minhngoc27169@gmail.com (N.P.-T.); 3 Center for Public Health and Ecosystem Research, Hanoi University of Public Health, Hanoi 10000, Vietnam; pdp@huph.edu.vn (P.P.-D.); ph.ntluong95@gmail.com (L.N.-T.); 4 Centers for Disease Control and Prevention, Atlanta, GA 30333, USA; jxn1@cdc.gov; 5 Federal Institute for Risk Assessment, National Laboratory for Trichinella, 10589 Berlin, Germany; anne.mayer-scholl@bfr.bund.de; 6 Working Group Meat Hygiene, Institute of Food Safety and Food Hygiene, Freie Universität Berlin, 14163 Berlin, Germany; maximilian.baumann@fu-berlin.de (M.B.); diana.meemken@fu-berlin.de (D.M.)

**Keywords:** *Trichinella* spp., *Taenia* spp., indigenous pig, seroprevalence, risk factors, Vietnam

## Abstract

Trichinellosis and cysticercosis remain challenges to human health and animal productivity worldwide, especially in developing countries. While information on the occurrence of both diseases is infrequent, they are endemic in parts of Vietnam and mainly related to indigenous pigs kept by ethnic minorities. This study aimed to determine the seroprevalence and risk factors of both diseases in indigenous pigs and explore the perception and awareness of both human and pig trichinellosis and cysticercosis of pig farmers. A total of 352 pig sera samples from 131 holdings were collected and analyzed using ELISA antibody tests in six communes in the Da Bac districts of Hoa Binh province, Vietnam. A survey was conducted with representatives from these households to understand the knowledge and perspective on food-borne parasitic diseases. Overall, the seroprevalence of trichinellosis and *T. solium* cysticercosis was 13.6% (95% CI 10.2–17.7) and 1.7% (95% CI 0.6–3.7), respectively. The seroprevalence of trichinellosis was significantly higher in female and older pigs. Risk perception and knowledge of interviewed people on both human and pig trichinellosis and cysticercosis of pig farmers was poor. Risky practices, including free roaming of pigs and eating undercooked or fermented pork, were observed. Educational and awareness campaigns aligned with further research on feasible practice changes are critical to addressing these issues.

## 1. Introduction

Trichinellosis and cysticercosis are the most widely distributed zoonotic parasitic diseases in the world and ranked first and seventh in terms of burdens on public health, socio-economic and trade impacts [1,2]. Cysticercosis and trichinellosis are neglected zoonotic diseases characterized by a variety of clinical signs in humans, including epilepsy as a manifestation of cysticercosis, and muscle pain with fever for trichinellosis [1,3]. Both result in pork being rejected during meat inspection, thus impacting the economy and nutrition. Porcine cysticercosis occurs due to exposure to eggs from feces of infected people in the environment [1,4]. *Trichinella* infection occurs when pigs ingest rodents or other wildlife infected with *Trichinella* spp. [3]. Humans become infected with trichinellosis and *T. solium* taeniasis by consuming raw or undercooked pork containing *Trichinella* larvae or *T. solium* cysticerci. Human cysticercosis also occurs when people ingest eggs or proglottids of *T. solium* from contaminated vegetables or water [5]. Pork and related products remain the most predominant source of *Trichinella* and *T. solium* infections in humans, especially when pigs are raised under free-range or backyard production conditions [6]. Due to the multifactorial life cycles of these two parasites, the endemicity of food-borne parasitic diseases (FBPDs) in developing countries has been associated with inadequate sanitation, use of untreated human waste in agriculture, free ranging of pigs, poor knowledge about parasites and eating raw or undercooked pork [3,7,8].

It is estimated that 11 million people worldwide are infected with *Trichinella* spp., with significant under-reporting likely in many parts of the world [9]. *Trichinella* spp. has been found in domestic and wild animals in 66 countries, while human trichinellosis has been documented in 55 countries [2,10,11]. In southeast Asia, trichinellosis has been reported in Laos, Thailand, Cambodia and Vietnam, where the practice of eating undercooked or fermented pork is common [9,11,12]. From 1970 to 2012, in Vietnam, *Trichinella* spp. was diagnosed in at least five outbreaks in the northern mountainous provinces, affecting a total of 126 patients and causing 8 fatalities [13]. Among pigs, seroprevalence (antigen-ELISA) of trichinellosis was reported to be approximately 20% for the north of the country [14]. Indigenous pig is the predominant source of outbreaks of human trichinellosis in northern Vietnam, while wild boar consumption was reported in southern and central Vietnam [13,15].

When it comes to cysticercosis and taeniasis, approximately 50 million people are suffering from neurocysticercosis due to *T. solium* worldwide, with more than 50,000 deaths per year [2,16,17]. In Vietnam, human taeniasis has been reported in 50 of 63 provinces, while cysticercosis has been reported in 55. A study on human tapeworms in north Vietnam from 65 patients reported that *Taenia asiatica* was the most popular species (55.4%), followed by *T. saginata* (38.5%) and *T. solium* (6.2%) [18]. The percentage of people infected with the tapeworm *T. solium* also varied from 0.1 to 12.0% depending on the area, and almost all reported cases were associated with consumption of raw pork [19,20,21]. In pigs, there are fragmented and out-of-date data since 2000 on the prevalence of porcine cysticercosis. Some recent surveys were carried out mainly in the northern mountainous regions among the ethnic minorities who practice free-roaming pig husbandry. Apparent cysticercosis seroprevalence using Ag-ELISA found in pigs was 9.91% [21], and other studies at pig slaughterhouses reported less than 0.06% of carcasses examined to be infected with *T. solium* [22,23]. In the central highlands of Vietnam, seroprevalence against *T. solium* cysticercosis in pig was 0.94%, using an enzyme-linked immunoelectrotransfer blot test (EITB) [24]. In addition, *T. hydatigena* prevalence reported in southern and northern Vietnam was 31.8% [25] and 25.1% [26], respectively, with low prevalence (0.5%) of *T. asiatica* cysticerci [27].

Hoa Binh province is located next to endemic provinces for trichinellosis (Dien Bien, Lai Chau, Son La). In Hoa Binh, indigenous pigs (Ban breed, a local breed of ethnic minorities in mountainous areas, characterized by black-color hair and skin, less interactive with commercial/exotic breeds [28]) are preferred by consumers because they consider this pork to have a better taste and to be “safe by nature”. Because of this, indigenous pork is often sold at a higher price. In addition, free ranging of indigenous pigs is a common style in Hoa Binh, often practiced by ethnic minorities who have low educational level [29,30]. The consumption of undercooked or fermented pork (a traditional dish named “Tap”) is observed in the area and carries the risk of FBPDs infection for local people. Trichinellosis, cysticercosis and taeniasis are often used to illustrate the One Health application in Vietnam, in addition to five key zoonotic diseases defined by both Vietnam Ministries of Health and Agriculture. While available studies have been conducted mainly in endemic provinces, this study aimed to determine the seroprevalence and risk factors of both diseases in indigenous pigs and explore the perception and awareness of human and pig trichinellosis and cysticercosis of farmers. The information is expected to contribute to more targeted planning of parasite control measures in the area to promote socio-economic development through pig production.

## 2. Materials and Methods

### 2.1. Study Sites and Design

**Figure 1:**
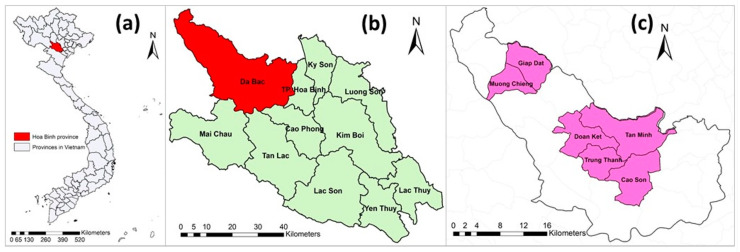
(**a**) Map of Vietnam with Hoa Binh province indicated; (**b**) Hoa Binh province and its districts; and (**c**) Da Bac district with six studied communes (pink).

Hoa Binh province is located in the northern region of Vietnam. Hoa Binh was selected because of its high proportion of ethnic minorities (accounting for 74% of the province’s population) [29] who often raise indigenous pigs under free-range management systems. The Da Bac district was chosen because of its highest indigenous pig population in Hoa Binh province. Da Bac is a mountainous district, has a population of approximately 60,000 (in 2017) and consists of 1 town and 19 communes. Out of 20 towns and communes, 6 communes—namely Cao Son, Doan Ket, Giap Dat, Muong Chieng, Tan Minh and Trung Thanh—were purposely selected based on their highest number of Ban pigs (Figure 1).

A cross-sectional study was conducted between June and August 2018 in six selected communes. Jugular vein blood of pigs was sampled, and a survey on the knowledge and awareness of pig owners about food-borne parasitic diseases was conducted in the same sampled pig household. Households were selected randomly from an existing list of all households with pigs in the commune. The household list was provided by local veterinary services in the respective communes, and a selection of participating households was compiled based on their consent. When a household disagreed to join, another one was selected from their eligible neighborhood.

### 2.2. Sample Size

The sample size was calculated based on the following assumptions: 5% expected prevalence for both diseases, 95% level of confidence, 2.5% precision and an intra-cluster correlation of 0.2. A total of 352 pigs were calculated and sampled. Proportional sampling was applied to determine the number of pigs to be sampled in each commune (Table 1).

### 2.3. Data Collection

#### 2.3.1. Pig Samplings and Observational Checklist

Up to three pigs aged above three months were randomly selected per household for (jugular vein) blood sampling, with the exclusion of breeding stock and pregnant sows. For each pig sampled, an observational checklist was applied to obtain basic information on age, sex, estimated weight, breed and raising system.

#### 2.3.2. Questionnaire

A structured questionnaire was developed and consisted of three parts: (i) general information of the household characteristics, such as age, gender, ethnicity, education level, household size and toilet type used; (ii) information on pig management, such as herd size, pig raising system, origin of pigs, sale and slaughter of pigs, the proportion of own pigs consumed, keeping other animals (cattle, buffalo, chicken, dog, cat) and the presence of rodents on farm; and (iii) knowledge and perception of respondents regarding food-borne parasitic diseases. A history of undercooked and fermented pork/pork product consumption was also recorded, with the frequency of consumption (daily, weekly, monthly or occasionally on special events, such as weddings, funerals and lunar new year holidays). After obtaining written consent from respondents, face-to-face interviews were conducted by trained researchers.

### 2.4. Data Management and Analysis

#### 2.4.1. Laboratory Testing

All pig serum samples were subjected to an in-house ELISA test, which was developed by the National Reference Laboratory for Trichinella, Federal Institute for Risk Assessment, (BfR), Germany, for detecting antibodies against *Trichinella*. Antibodies against cysticercosis were detected by EITB assay that used lentil lectin-bound glycoproteins (LLGP) antigen with 99% for both specificity and sensitivity [31,32,33]. We set two levels of seroprevalence in this study: individual pig and farm levels. A farm was defined as positive if there was at least one pig that obtained a positive result for the respective tested diseases.

#### 2.4.2. On-Farm Questionnaire

Collected data from on-farm questionnaires were entered into spreadsheets (MS Excel, ver. 2016) and cross-checked by two independent researchers. For the Likert-scale question, “Strongly agree”, “Agree”, “Neutral”, “Disagree” and “Strongly disagree”, when a respondent indicated “Strongly agree” or “Agree” with a negative or “Undesirable” statement, the response was classified as “Undesirable”. The reverse was considered “Desirable”.

#### 2.4.3. Data Analysis

Data analysis was performed using R3.4.4 (R Core team 2018). Two data sets on the farm (household) and individual pig levels were analyzed for all pig blood samples and related information obtained from the sampling form, such as age, weight, pig raising system. Both univariate and multivariate analyses were performed using generalized linear mixed-effect models (GLMMs) in lme4 package [34] in R to test for association between seropositivity of the two diseases and the explanatory variables. Two separate GLMMs models for the farm (household)- and (individual)-pig-level were used, and the outcome variables were seropositive or seronegative status, at farm (at least one pig testing positive was assigned a positive farm, otherwise negative) and individual pig level, respectively. The commune was set as a random effect in herd-level GLMM, whereas the commune and household were set as random effects in individual pig-level GLMM to control confounding bias. The variables used in univariate analysis were gender, age, the weight of the pig, farm scale, pig raising system, toilet type at the farm, recent deworming of pigs and presence of rodents at the farm. Variables that had *p*-value ≤ 0.20 in the univariate analyses were then included in the multivariate analysis. Model simplification, using backward stepwise, was performed to determine the risk or preventive factors. The significance level was set at *p* ≤ 0.05. The results were reported as adjusted odds ratios with 95% confidence intervals (CIs).

## 3. Results

### 3.1. Seroprevalence of Pig Trichinellosis and T. solium Cysticercosis in Pigs

In total, 352 pigs were sampled with a mean age of 8 months (range: 3 to 72 months). Male and female pigs accounted for 58.2% (205 pigs) and 41.8% (147 pigs), respectively. Forty-eight (13.6%; 95% CI: 10.2–17.7) and six (1.7%; 95% CI: 0.6–3.7) pigs were found seropositive with trichinellosis and cysticercosis, respectively. Positive responses for *Trichinella* spp. were found across all six selected communes, whereas seropositivity for pig *T. solium* cysticercosis was only detected in three communes (Cao Son, Giap Dat and Doan Ket). While there were some variations of seroprevalence of *Trichinella* spp. and pig *T. solium* cysticercosis occurrence in the studied communes, no significant difference was found. Only one pig in Giap Dat commune was seropositive for both porcine trichinellosis and *T. solium* cysticercosis (Table 2).

### 3.2. Risk Factors Related to Seroprevalence of Trichinellosis and T. solium Cysticercosis in Pigs

#### 3.2.1. Pig Raising Practice and Sanitation Conditions

The majority of households (102 out of 131, 77.9%) kept fewer than 10 pigs in their home, while 29 households (22.1%) had 10–50 pigs. Only one household had 100 pigs. Sixty-five percent of households (86/131) reported that they allowed their pigs to freely scavenge in the surrounding village.

All pigs in the interviewed households were indigenous pigs, which were mostly bought in the same commune. Almost all pigs were sold at the same village (126 households, 96%) and slaughtered at their home (129, 98%) before being sold at the market. Farmers raised pigs mainly for sale (81 households, 61.8%). Within those pig-selling households, 70% of pigs were sold, while the rest were used for home consumption.

Over 82% (108/131) of respondents reported that they used antiparasitic drugs (such as Ivermectin (0.6%) or Mebendazole (10%) and commercial name Tayzu/tay giun san) for their pigs, among which 92.6% (100/108) used them within the last three months. Respondents who did not use antiparasitic drugs (18 out of 23) stated that it was not necessary (18 respondents) or too costly (5 respondents).

Out of 131, 112 households (85.5%) reported that rodents were found near houses and farms. The proportion of participants having pit latrines and flush toilets in their houses was 84.7% (111 households) and 15.3% (20 households), respectively.

#### 3.2.2. Univariable Analysis

Table 3 shows the univariate GLMM analysis results. Several factors, such as weight of pigs, pig raising system, toilet types used and keeping dogs at the farm, showed a tendency of associations (however, at *p* < 0.1), while sex and age were significantly associated (*p* < 0.05) with *Trichinella* spp. seropositivity. The same univariate GLMM model was applied for *T. solium* cysticercosis seropositivity; however, no association was found.

#### 3.2.3. Multivariable Analysis

Multivariable GLMM results showed that there were statistically significant differences in *Trichinella* seroprevalence among the age groups of pigs. Pigs aged 12 months and above were more likely to be seropositive than younger pigs. The other variables included in the multivariable GLMM analysis were not significantly associated with *Trichinella* spp. seropositivity in pigs. None of the selected variables in multivariable GLMM were associated with *T. solium* cysticercosis seropositivity in pigs.

### 3.3. FBPDs Knowledge and Attitude and Behaviour of Eating Raw or Undercooked Pork

#### 3.3.1. Demographic Information of Respondents

Out of 131 interviewed households, almost all respondents belonged to an ethnic minority in the following order: 87% were Tay, 10.7% Dao, 1.5% Muong and 0.8% Kinh. Of the total, 30.5% were female and 69.5% male. The average age of respondents was 44, with a range between 18 and 65 years, 46% of whom were aged between 31 and 45 (Table 4). The majority (69.5%) of interviewees had a secondary or lower education. More female respondents as compared to male respondents had only primary school education; only a few female respondents had access to advanced education (high school or higher).

#### 3.3.2. Knowledge

Overall knowledge on food-borne parasite diseases was poor. Only 18 out of 131 respondents reported that they had known at least one food-borne parasite disease. Interestingly, only 5 (out of 18) were able to name cysticercosis and 2 (out of 18) named trichinellosis. Three respondents reported that eating is the route of transmission, but none of them were able to specify the type of food that can cause the diseases. The rest (11) mentioned leptospirosis, streptococcosis, *E. coli* and foot-and-mouth disease.

#### 3.3.3. Attitude

**Figure 2:**
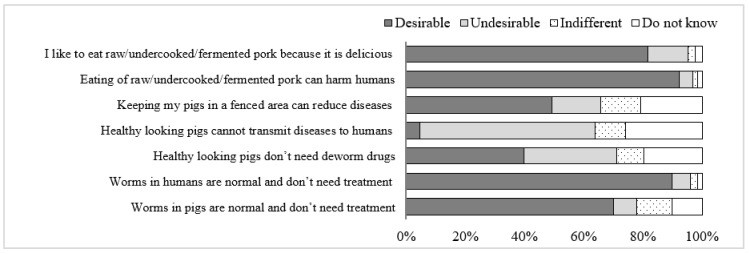
Attitudes of participants on FBPDs risk, prevention and treatment in Da Bac district, Hoa Binh province, Vietnam.

Results revealed that the majority of respondents had “Desirable” attitudes toward the prevention of FBPDs. In contrast, an “Undesirable” attitude was observed for the statements “Healthy-looking pigs do not need deworm drugs” and “Healthy-looking pigs cannot transmit diseases to humans” (Figure 2).

#### 3.3.4. Behavior of Eating Undercooked or Fermented Pork

A total of 37 out of 131 participants (28.2%) reported that they had consumed undercooked pork at least once since last year. None of them consumed undercooked pork daily, but weekly (2/131), monthly (5/131) and especially during cultural events (30/131). In addition, no significant difference was found between males and females in the frequency of consuming raw pork (*p* > 0.05, Fisher exact test).

## 4. Discussion

This study identified seroprevalence and risk factors of trichinellosis and cysticercosis in indigenous pigs and determined the perception and awareness of FBPDs of indigenous pig farmers. Seroprevalence of trichinellosis (13.6%) and *T. solium* cysticercosis (1.7%) indicated endemicity in trichinellosis and sporadic occurrence in *T. solium* cysticercosis in pigs in the studied communities. The interviewed community members demonstrated a lack of basic knowledge on zoonoses, especially FBPDs.

Several previous studies in Vietnam focused on the prevalence of trichinellosis, taeniasis and cysticercosis in humans and animals. Although trichinellosis outbreaks in humans occurred sporadically in some northern provinces of Vietnam, there was limited information on the prevalence in pigs in those provinces. In 2008, when a trichinellosis outbreak occurred and affected 22 people, resulting in 2 deaths in Bac Yen district, Son La province and a survey of 1035 pigs at the outbreak area found 205 positive cases, accounting for 19.9% [35,36]. In 2012, researchers reported a lower overall seroprevalence of 5.6% in other different districts of the province [14]. The survey in 2008 was conducted after a recent outbreak in humans, with an observed higher prevalence compared to 2012. Our finding on trichinella seroprevalence was lower than the two studies conducted in Son La province and higher than the study in Cambodia (2.5%) [37], but it aligns with reports for two provinces in Laos (9.3 and 14.4%) [38].

Seroprevalence of *T. solium* cysticercosis (1.7%) in pigs from this study is lower than findings from south and central Cambodia, which showed an overall rate of 4.7% testing positive for antigen-ELISA of *Taenia* spp. (*T. solium*, *T. asiatica* or *T. hydatigena*), with 7.6% for smallholders [39]. A higher cysticercosis seroprevalence was reported in four northern provinces of Laos, when 404 (68.5%) pigs were positive using antigen-capture ELISA, and the estimated true prevalence of pig *T. solium* cysticercosis with increments of test sensitivity (assuming specificity of 100%) varied from 0.8 to 8.5% [40]; meanwhile, in Cambodia (11.2%, antigen ELISA) [37] and in Myanmar (15.9%, antibody-ELISA) [41]. However, when comparing to other in-country surveys, the infection rate of cysticercosis in pigs in this study is similar. A report of porcine cysticercosis seroprevalence using EITB in 1281 local pigs in central highlands of Vietnam was 0.9% (12 out of 1281) [24]. In our survey, we used test methods developed by CDC (LLGP and recombinant EITB) to address the known low specificity of currently available commercial ELISA tests (e.g., apDia Cysticercosis AG ELISA), which are only genus specific [42]. The CDC LLGP and recombinant EITB are antibody detection assays, which are highly sensitive and specific and would not overestimate the prevalence. So, LLGP and recombinant EITB do not cross-react with *T. asiatica* or *T. hydatigena*. The drawbacks of antibody detection assays would be that antibodies detection may not be an indication of current infection, may be passive immune response for young piglets or exposure. Additionally, it may be possible that there are co-infections [27,33]. In humans, among 65 Vietnamese patients of taeniasis, the proportion of *Taenia asiatica*, *T. saginata* and *T. solium* was 55.4%, 38.5% and 6.2%, respectively [18]. The human tapeworm *T. solium* prevalence in Vietnam also varied from 0.1 to 12.0% depending on the area [19,20,21], and between 2006 and 2011, an average of 250 to 400 patients from 34 provinces in north Vietnam were hospitalized and treated for *T. solium* cysticercosis annually [43].

Multivariable analysis confirmed that seropositive trichinellosis in pigs increases by age. This result is similar to other reports in Son La province, Vietnam [35]. This is not surprising, as with the increasing age of the pigs, a longer exposure time is expected, explaining a typical cumulative effect for *Trichinella* infection. Univariable analysis indicated that male pigs have less chance of becoming infected (OR = 0.46, 95% CI = 0.32–0.68), which was also reported in a previous study in Son La [35]. In our study areas, the roaming of sows was a common practice, which could lead to higher exposure. Although the presence of rodents was not associated with *Trichinella* infection in our study, other authors found some causal linkages [44,45]. Rodents could contribute to the spread of *Trichinella* everywhere in the environment and potentially lead to exposure in humans. Moreover, it is validated that pig confinement systems can reduce the prevalence of zoonotic and internal parasite burdens in pigs [37,46,47]. In our survey, we observed a similar trend of increasing seroprevalence in unconfined pigs; however, it was not statistically significant.

In our survey, consuming undercooked or fermented pork mainly occurred on special events, as stated by 22.9% of respondents. There are limited data to understand the knowledge and perception of people about both trichinellosis and cysticercosis in humans and pigs. In this study, the interviewed community members demonstrated a lack of basic knowledge on zoonoses, especially trichinellosis and cysticercosis. They were unable to name either trichinellosis or cysticercosis in pigs or humans and demonstrated lack of understanding concerning the risk of contracting trichinellosis or taeniasis from eating raw fermented or undercooked pork. Their poor knowledge may have contributed to their practice of eating raw or undercooked pork, although occasionally. It was confirmed that raw and/or fermented pork was the source of human infection for both trichinellosis and cysticercosis in several outbreaks in Laos [48,49,50], Thailand [12], and some northern and central provinces of Vietnam [51,52,53]. Our results in pigs for both trichinellosis and *T. solium* cysticercosis seropositivity demonstrate a possible exposure for humans. Therefore, in this study, the documented practice of consuming raw fermented or undercooked pork during ceremonies/parties and hand, water, vegetable sanitation, human fecal and toilet management by communities can pose a risk to humans trichinellosis and *T. solium* cysticercosis, respectively. In addition, the risk perception of local people varied for different topics, and there was also a difference between animal health and public health statements. People usually care more about human health than animal health.

Pig trichinellosis and *T. solium* cysticercosis are great examples of One Health approach that recognizes the close linkages between animal, human and environmental health, and how this approach can be used to address these diseases with the participation of animal and human health workers [54], or male raw pork eating behavior [37]. Our results indicate poor knowledge in communities for both trichinellosis and *T. solium* cysticercosis. Thus, public health workers should educate people about the clinical symptoms of both diseases so they can seek medical help and early treatment when they notice clinical signs and symptoms. Risky pig farming practices, such as free roaming, are still used by some producers. This requires further promotion of good husbandry practices for raising pigs, such as the recently introduced Vietnamese Good Agricultural Practices (VietGAP). To gain higher compliance from pig farmers, this effort might include other pig diseases, such as hog cholera, foot-and-mouth disease (through vaccination) or recent African swine fever (through better biosecurity), as demonstrated by Okello et al. [55]. Here, educational campaigns are recommended to improve environmental hygiene in order to limit the risk of animal to human transmission of diseases. The public health sector has more information, but it is mainly based on passive outbreak reporting or research conducted following those outbreaks. Studies linking pig exposure and human outbreaks in affected communities are widely lacking but are critical to understanding the epidemiology and underlying risk factors, including control options for both trichinellosis and *T. solium* cysticercosis.

The results of this study should be interpreted considering both its strengths and limitations. The latter could have been affected by inaccurate recall and confirmation bias in survey answers. To limit these biases, the research team was trained, and discussions took place after each data collection day to ensure similar viewpoints or address other issues or problems. While the study followed a random sampling approach, communes were selected purposively, following predefined criteria. Hence, conclusions for other areas should be drawn with caution. We anticipate that data from this project will contribute to broaden the national seroprevalence database to substantiate interventions in Hoa Binh province and reduce the prevalence of pig trichinellosis and *T. solium* cysticercosis in Hoa Binh and other similar communities in Vietnam. These findings have important implications for developing appropriate intervention strategies for reducing zoonotic parasitic diseases infection in pigs, as well as support the improvement of awareness and knowledge on those diseases among local people, public health practitioners and veterinary service providers in the provinces.

## 5. Conclusions

This study demonstrated a current endemicity of trichinellosis and sporadic occurrence of *T. solium* cysticercosis infection in the pig population in Hoa Binh province. Both diseases still pose a public health risk to communities. This is of even greater importance, as people’s awareness and knowledge of these diseases is insufficient, and risky practices, such as free roaming of pigs or eating raw fermented and undercooked pork, are still occurring. Therefore, awareness-building campaigns about these diseases and the risks they pose to human and animal health need to be conducted in this area, including improving sanitation conditions, using pig confinement systems, better management of both human and animal feces, such as using closed-pit latrines. A One Health framework should be followed to implement these approaches in collaboration with veterinarians, medical doctors, researchers, relevant governmental agencies and communities.

## Figures and Tables

**Table 1:** Number of sampled pigs and interviewed households in Da Bac district, Hoa Binh province, Vietnam.

Commune	Number of Indigenous Pigs	Number of Pigs Sampled	Number of Households Interviewed
Cao Son	1001	76	26
Giap Dat	890	68	24
Tan Minh	790	60	24
Doan Ket	787	60	23
Muong Chieng	647	50	18
Trung Thanh	494	38	16
Total	4609	352	131

**Table 2:** Seroprevalence of pig trichinellosis and *T. solium* cysticercosis by household and individual pig levels in different studied communes.

Commune	Number of Households *			Number of Pigs		
	N	Seropositive with *trichinellosis* *n* (%)	Seropositive with *T. Solium* Cysticercosis *n* (%)	N	Seropositive with *trichinellosis* *n* (%)	Seropositive with *T. Solium* Cysticercosis *n* (%)
Tan Minh	24	11 (45.8)	0 (0)	60	13 (21.7)	0 (0)
Cao Son	26	9 (34.6)	1 (3.8)	76	10 (13.2)	1 (1.3)
Giap Dat	24	8 (33.3)	2 (8.3)	68	10 (14.7)	2 ** (2.9)
Doan Ket	23	5 (21.7)	2 (8.7)	60	7 (11.7)	3 (5.0)
Muong Chieng	18	5 (27.8)	0 (0)	50	5 (10.0)	0 (0)
Trung Thanh	16	2 (12.5)	0 (0)	38	3 (7.9)	0 (0)
Total	131	40 (30.5)	5 (3.8)	352	48 (13.6)	6 (1.7)

(*) Seropositivity at household (farm) level. (**) One pig was seropositive for both trichinellosis and *T. solium* cysticercosis.

**Table 3:** Univariate GLMM results at individual pig- and farm-levels related to *Trichinella* spp. seropositivity in Da Bac district, Hoa Binh province, Vietnam.

	Individual Pig Level (*n* = 352)			Pig Farm Level (*n* = 131)		
Variable	Number of Positive Pigs/*n*	OR_adj_ (95% CI)	*p*-Value	Number of Positive Farms/*n*	OR_adj_ (95% CI)	*p*-Value
Sex of pigs						
Female	35/205	Ref	-			
Male	13/147	0.5 (0.2–0.9)	**0.04**			
Age groups						
≤6 months	19/199	Ref	-			
7–12 months	16/105	1.8 (0.8–4.1)	**0.18**			
>12 months	13/47	4.2 (1.6–11)	**0.003**			
Weight of pigs						
≤15 kg	22/209	Ref				
>15 kg	26/143	2.0 (1.0–4.0)	**0.07**			
Farm scale						
≥10 pigs/farm	12/84	Ref	-	29/102	Ref	-
<10 pigs/farm	36/268	0.9 (0.4–2.3)	0.84	11/29	0.65 (0.42–1.01)	0.33
Pig raising system						
Fenced	15/123	Ref	-	12/45	Ref	-
Free roaming	11/53	2.2 (0.7–6.6)	**0.18**	9/21	2.06 (1.18–3.59)	**0.19**
Semi	22/176	1.0 (0.4–2.5)	0.92	19/65	1.14 (0.74–1.75)	0.76
Toilet type						
Pit latrine	36/297	Ref	-	31/111	Ref	-
Flushing	12/55	2.3 (0.9–6.1)	**0.09**	9/20	2.17 (1.3–3.63)	**0.13**
Pig dewormed recently						
>1 month	31/215	Ref	-	35/108	Ref	-
≤1 month	17/137	0.8 (0.4–1.8)	0.64	5/23	1.73 (1.00–2.99)	0.32
Keeping dog at farm						
No	7/86	Ref	-	6/33	Ref	-
Yes	41/266	2.1 (0.8–5.8)	**0.13**	33/98	1.90 (1.17–3.08)	**0.18**
Keeping cat at farm						
No	24/192	Ref	-	17/57	Ref	-
Yes	24/160	1.3 (0.6–2.7)	0.55	23/74	1.21 (0.82–1.77)	0.62
Presence of rodents at farm						
No	3/34	Ref	-	3/12	Ref	-
Yes	42/299	1.7 (0.4–7.9)	0.47	34/112	1.35 (0.66–2.75)	0.67
Household members consume raw pork *						
No	33/250	Ref	-	28/94	Ref	-
Yes	15/102	1.1 (0.5–2.6)	0.79	12/37	1.14 (0.74–1.75)	0.76

CI: Confidence interval; Ref: Reference; *p*-values in bold: variables (with *p* ≤ 0.2) were selected for multivariable analysis; * Undercooked and/or fermented pork.

**Table 4:** Demographic information of respondents.

Characteristics	Male (*n* = 91)		Female (*n* = 40)		Total (*n* = 131)	
	*n*	%	*n*	%	*n*	%
Age group						
18–30	10	11.0	8	20.0	18	13.7
31–45	45	49.4	16	40.0	61	46.6
46–60	32	35.2	8	20.0	40	30.5
>60	4	4.4	8	20.0	12	9.2
Ethnic group						
Tay	81	89.0	33	82.5	114	87.0
Dao	9	9.9	5	12.5	14	10.7
Muong	0	0	2	5.0	2	1.5
Kinh	1	1.1	0	0	1	0.8
Occupation						
Farmer	90	99.0	39	97.5	129	98.5
Other	1	1.0	1	2.5	2	1.5
Education						
Primary school or less	24	26.4	17	42.5	41	31.3
Secondary school	34	37.4	16	40.0	50	38.2
High school or higher	33	36.2 ^a^	7	17.5 ^b^	40	30.5

^a^, ^b^*p* < 0.05.

## Data Availability

Data will be provided by authors upon request.
